# Prospective selected biomarkers in COVID-19 diagnosis and treatment

**DOI:** 10.2217/bmm-2021-0038

**Published:** 2021-09-20

**Authors:** Tahani Tabassum, Ahsab Rahman, Yusha Araf, Md A Ullah, Mohammad J Hosen

**Affiliations:** ^1^Department of Mathematics & Natural Sciences, Biotechnology Program, School of Data & Sciences, Brac University, Dhaka, Bangladesh; ^2^Department of Genetic Engineering & Biotechnology, School of Life Sciences, Shahjalal University of Science & Technology, Sylhet, Bangladesh; ^3^Department of Biotechnology & Genetic Engineering, Faculty of Biological Sciences, Jahangirnagar University, Dhaka, Bangladesh

**Keywords:** biomarkers, COVID-19, detection, prognosis, pulmonary infections, stratification

## Abstract

COVID-19 has become a global health concern, due to the high transmissible nature of its causal agent and lack of proper treatment. Early diagnosis and nonspecific medical supports of the patients appeared to be effective strategy so far to combat the pandemic caused by COVID-19 outbreak. Biomarkers can play pivotal roles in timely and proper diagnosis of COVID-19 patients, as well as for distinguishing them from other pulmonary infections. Besides, biomarkers can help in reducing the rate of mortality and evaluating viral pathogenesis with disease prognosis. This article intends to provide a broader overview of the roles and uses of different biomarkers in the early diagnosis of COVID-19, as well as in the classification of COVID-19 patients into multiple risk groups.

COVID-19 outbreak has become a global public health issue and brought the world to a standstill [1]. Emerging from Wuhan, People's Republic of China, in December 2019, COVID-19 has affected more than 46 million people in 218 countries, and more than one and a half million people have died due to this disease till the date of this writing [2]. COVID-19 is caused by SARS-CoV-2, which is a positive, single-stranded, enveloped RNA virus from the family Coronaviridae [3]. With the help of spike protein (S-protein), SARS-CoV-2 invades the host cell by interacting with the ACE-2 receptor on the host cell membrane [4,5]. Due to its contagious characteristic, it is compulsory to ensure early stratification of SARS-CoV-2-infected patients [6]. The symptom of COVID-19 is heterogeneous, but the common symptoms include fever, dry cough and respiratory distress [7,8]. Many symptoms overlap with those of common flues, making it hard to understand the pathomechanism and diagnosis, as well as the treatment of this disease. Moreover, the lack of sustainable therapeutics is a treatment challenge for COVID-19. The identification of a perfect biomarker set for COVID-19 will enable clinical studies to determine whether a therapeutic has a clinically significant effect on its phenotype [9–12]. In addition to that, biomarkers can play a significant role in the early diagnosis of COVID-19, effectively differentiating it from other pulmonary infections. Pulmonary infections are infections that cause lung inflammation, thereby damaging their larger airways and smaller air sacs. Oftentimes, the overlapping symptoms of COVID-19 with other pulmonary infections such as pneumonia make it difficult to identify COVID-19 patients [13,14]. In this review, we will provide comprehensive insights into the role of different biomarkers for detecting COVID-19 as well as the factors differentiating it from other pulmonary infections.

## Pathophysiology of COVID-19

**Figure 1.**
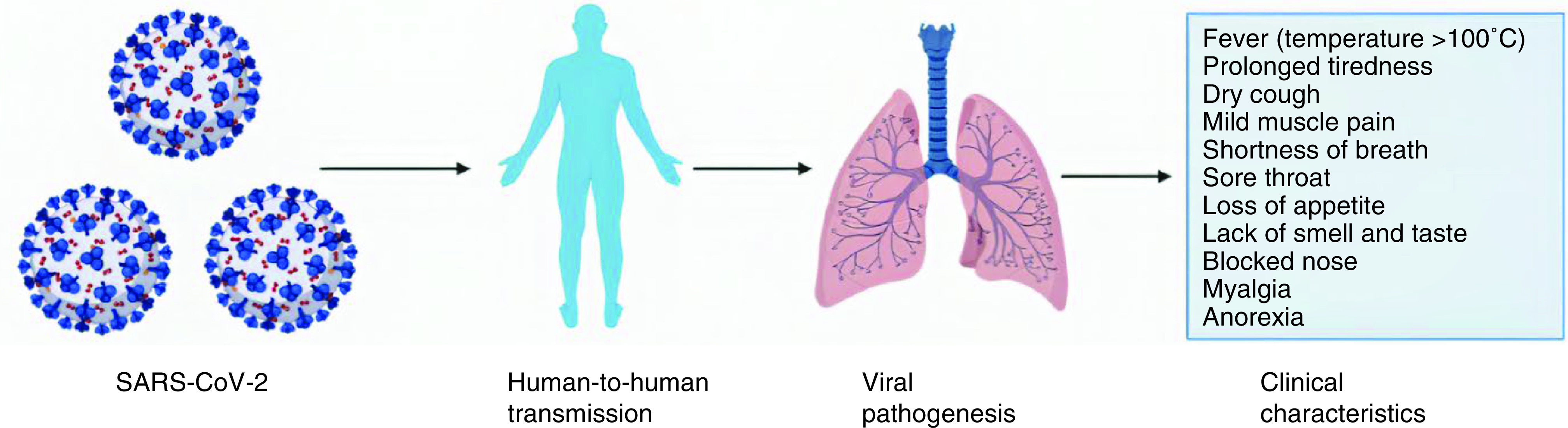
Clinical courses of SARS-CoV-2. The virus enters the body and damages the lungs. Symptoms may vary from person to person.

SARS-CoV-2 follows a lytic cycle to replicate itself by employing the metabolic machinery of a cell. It invades a human host cell through five significant steps – attachment, penetration, biosynthesis, maturation and release (Figure 1) [15,16]. There is a molecular key named ‘spike protein’ (S-protein) in SARS-CoV-2 which provides a route to the virus to enter the cell. Using the S-protein, the virus attacks the host cell by interacting with ACE-2 receptors [17]. There is a furin cleavage site in S-protein, which is responsible for the robust affinity of ACE-2 toward SARS-CoV-2. Furin is present in numerous body organs including the lungs, liver and small intestine, which indicates that the virus can infect multiple organs of the body [18]. Another potential entryway may come via its association with CD147, a transmembrane glycoprotein expressed in high levels in pathogen-infected cells and tumor tissues [19].

Once entered, the viral genome is released, which ultimately leads to the translation of viral polymerase protein. After the translation process, RNA replication takes place. It is then followed with subgenomic transcription and translation of viral structural proteins. S-protein, membrane protein and envelope protein combined with the nucleocapsid. Ultimately, mature virion is formed and exocytosis occurs [20].

A cytokine storm – a physiological phenomenon where the immune system releases excessive level of cytokines – can be observed in COVID-19 following the release of genomic RNA into the cytoplasm. Toll-like receptors, such as TLR-3 and TLR-4, are triggered when double-stranded DNA induces an immune response. While TLR-3 uses a signaling pathway cascade to stimulate type-I interferon, TLR-4 recruits immune cells in the infection site through the activation of pro-inflammatory cytokines [21,22].

A cytokine storm is the result of a sudden acute increase in circulating levels of pro-inflammatory cytokines, including IL-6, IL-1, TNF-α and interferon. This ultimately leads to the influx of several immune cells like macrophages, T cells and neutrophils into the site of infection. Moreover, the elevated cytokine release, specifically the IL-6 stimulation, leads to an increased level of synthesis of prototypic acute phase reactant like C-reactive protein (CRP) from the liver, in the bloodstream. Eventually, all these events lead to different sequalae like destabilization of endothelial cell-to-cell interaction, destruction of capillary and vascular barrier and damage of multiple organs and tissues, which may ultimately lead to the demise of the infected individual [23].

Another potential receptor for viral infection is DC-SIGN, expressed highly in dendritic cells (DCs) and macrophages. However, further studies need to be conducted for it to be confirmed [24].

## Differential biomarkers between SARS-CoV-2 & other pulmonary infection patients

A clinical presentation of COVID-19 has several overlapping features with other pulmonary infections including pneumonia, chronic bronchitis and so on (Table 1). Pneumonia patients exhibit symptoms like high fever, cough, fatigue, lack of appetite, etc. [13]. The clinical presentation of chronic obstructive pulmonary disease (COPD) includes cough, dyspnea and extreme tiredness [25]. Similar symptoms can be visualized in chronic bronchitis as well [26]. All these pulmonary diseases have signs and symptoms that resemble the signs and symptoms of COVID-19. As a result, oftentimes it becomes difficult to distinguish between SARS-CoV-2-infected patients and other pulmonary disease patients. Biomarkers have proven to be effective in differentiating between these two classes of patients [14,27–29].

**Table 1.** Overlapping symptoms between COVID-19 and other pulmonary diseases.

Disease	Fever	Dry cough	Headache	Dyspnea	Fatigue	Lack of appetite	Ref.
COVID-19	Yes	Yes	Yes	Yes	Yes	Yes	[5,7,8]
Pneumonia	Yes	Yes	No	Yes	Yes	Yes	[13,27]
Chronic bronchitis	No	Yes	No	Yes	Yes	No	[26,28]
COPD	No	Yes	Yes	Yes	Yes	Yes (sometimes)	[25,29]

COVID-19 patients had a lower proportion of basophils (BASP), lymphocyte count (LYM) and white blood cell count (WBC) compared with the patients with other pulmonary diseases [13]. The most discriminating biomarkers between these two groups were BASP, prothrombin time activity (PTA), prothrombin time (PT) and international normalized ratio (INR). COVID-19 patients had relatively lower PT and INR and relatively higher PTA. A lower proportion of basophils represent COVID-19 patients, indicating its role in producing T-lymphocyte. Therefore, decreasing basophils may result in the disruption of the adaptive immune system. As a result, it is a crucial biomarker to differentiate between the two groups of patients [30,31].

It was also found that some of the coagulation parameters were different between these two groups as well. COVID-19 patients having relatively lower PT and INR and relatively higher PTA indicate that they were subjected to higher coagulation. Higher coagulation has the potentiality to blunt inflammation, which could explain why clinical inflammation symptoms are not obvious in COVID-19 patients [30,32,33]. So, it is evident that biomarkers can play a salient role in distinguishing COVID-19 patients from other pulmonary disease affected patients.

## Differential expression of different biomarkers & their possible mechanism in COVID-19 patients

### Procalcitonin

Procalcitonin (PCT) is the precursor of the hormone calcitonin, involved in regulating the levels of calcium and phosphate in the bloodstream [34]. PCT has been beneficial for the diagnosis of severe bacterial infections, as well as for guiding antimicrobial stewardship [35]. PCT is also known as ‘hormokine’; either follows a hormonal expression pathway or a cytokine-like expression pathway in response to bacterial infections [36].

The increased concentration of IFN-γ in response to viral infections can inhibit the production of PCT. It is therefore expected that PCT values should remain within the minimum range in patients with noncomplicated COVID-19 infection [37]. Nevertheless, several studies reported an elevated level of PCT among severe COVID-19 patients [38]. Several reasons might be attributable to the increased PCT levels with disease progression. Due to viral-induced lung tissue damage, the normal bacterial flora might become invasive leading to the development of secondary bacterial pneumonia and enhanced PCT secretion. Another reason might be bacterial translocation in response to inflammation and septic illness induced by COVID-19-associated pneumonitis that can significantly increase PCT secretion [39]. Although in mild cases the level of PCT is within the normal range, the levels increase once the disease starts becoming complicated. This substantial increase in the PCT level reflects bacterial co-infection, which can be a significant indicator of disease severity [38]. Several other studies have reported that analysis of PCT levels can be positively associated with the severity of COVID-19 infection (Table 2) [40–45].

**Table 2.** Range of biomarkers in mild and severe COVID-19 patients.

Biomarker	Range in normal human	Range in mild COVID-19	Range in severe COVID-19	Ref.
Procalcitonin (ng/ml)	0.45	0.05 ± 0.05	0.44 ± 0.55	[44,45]
C-reactive protein (mg/l)	<10	33.2	57.9	[46,47]
Serum amyloid A (mg/l)	<10	89.78 ± 54.75	144.29 ± 57.33	[48,49]
IL-6 (pg/ml)	0–16.4	<80	>80	[50,51]
Lymphocyte count (cells/μl)	1000–4800	1.4 ± 0.15 × 10^3^	0.8 ± 0.11 × 10^3^	[52,53]
Platelet count	125 × 10^9^–350 × 10^9^/l	131 × 10^9^–263 × 10^9^/l	165 × 10^9^–263 × 10^9^/l	[54,55]
D-Dimer (mg/l)	<0.25	0.5	2.4	[56,57]
Lactate dehydrogenase (U/l)	140–280	151	248	[58]
Cardiac troponin (ng/ml)	<0.04	0.03–0.09	>0.09	[59,60]
Serum ferritin (ng/ml)	12–300	291.13	1006.16	[61,62]

### C-reactive protein

C-reactive protein (CRP), an acute-phase inflammatory protein synthesized primarily by the liver, plays a crucial role in the innate immunity. CRP is a significant indicator of severity in COVID-19 [63]. In normal conditions, the concentration of CRP in the blood is less than 10 mg/l [64], but the concentration may increase up to 1000-fold in response to severe bacterial infections and tissue damage [65]. This rapid increase in CRP concentration is observed during the initial 6–8 h and gives the highest peak in around 48 h after the disease onset [64]. However, once the stimuli end, the concentration keeps on decreasing exponentially [66]. Once the conditions are resolved and CRP concentration falls within a range, it can serve as a useful biomarker for estimating disease severity [64].

CRP levels are also reported positively correlated with lung lesion during the early stages of SARS-CoV-2 infection [66]. High CRP levels can also be used for early diagnosis of severe pneumonia in COVID-19 patients [67]. The effect of increased CRP in severe SARS-CoV-2 infections can be linked to the overproduction of inflammatory cytokines. Cytokine storm is a major mechanism of inducing lung damage by the SARS-CoV-2. These inflammatory cytokines and their corresponding tissue damage may evoke the overproduction of CRP and mark worse disease prognosis (Table 2) [46,47,68].

### Serum amyloid A

Serum amyloid A or SAA protein, synthesized primarily by the liver, plays an important role in the body's inflammatory responses and lipid metabolism. Amyloid A is also known for its function in central nervous system and its association with multiple neurodegenerative disease has already been postulated. However secreted small amount during the normal conditions of the body, but the SAA levels are elevated during the acute phase of an inflammatory disease due to the stimulated inflammatory mediators including IL-1, IL-6, TNF-α [69]. Thus, SAA can be used as an important and independent biomarker to detect both viral and bacterial infections.

Several studies have reported that SAA can be used as an independent predictive biomarker of COVID-19 [70,71]. An increased level of SAA has been found to be associated with the severity of COVID-19 infection [70]. SAA levels were found increased in severe patients and the expression was significantly higher in acute phase infection compared with the convalescent phase infection [72].

The precise mechanism for the elevated release of this acute-phase protein is still unclear, but could be influenced by the increased production and secretion of inflammatory mediators, a characteristic phenomenon of SARS-CoV-2 infection [7]. SAA was also found to be more sensitive and efficient in detecting minor inflammatory stimuli in several viral infections than CRP (Table 2) [48,49,73]. Based on such observations, it is clear that SAA can be a vital biomarker to predict severe outcomes of COVID-19. Further evidence needs to be accumulated to better understand the correlation between SAA levels and disease severity in COVID-19 patients.

### Interleukin-6

IL-6 is one of the most important cytokines that is produced in response to infections and tissue damages and plays a crucial role in the host immune defense mechanism. It is mostly synthesized during the initial stage of inflammation and then moves to the liver to rapidly induce the secretion of a huge range of acute-phase proteins [74]. As a major mediator of the inflammatory response, it has been found to play a pivotal role in the pathophysiology of COVID-19-induced lung damage [75]. Besides, levels of IL-6 are reported to be linked with the increased need for incubation and mechanical ventilation of COVID-19 patients [76,77].

Multiple studies have reported a significant difference in the IL-6 levels between complicated and non-complicated COVID-19 cases. It was reported that IL-6 levels were around three-times higher in the severe COVID-19 patients than the non-severe patients [64,66]. Higher IL-6 levels could also be linked to higher mortality rates [77]. Thus, IL-6 can be a good prognosticator for combined end-point progression to detect severe COVID-19 infection and in-hospital mortality rate detection [75].

Acute respiratory distress syndrome (ARDS) generates a cytokine storm by eliciting the overproduction of many inflammatory mediators including IL-6, and contributes to the mortality and severity of a COVID infection. In COVID-19 patients, the overproduction of IL-6 was found to be linked with complicated outcomes, such as hepatic, renal, cardiac and lung injuries [78]. At present, several IL-6 blocking agents are being used to treat COVID-19 patients and are displaying positive outcomes by reducing disease severity (Table 2) [50,51,79]. It is suggested that further investigation be initiated, regarding the reliability of IL-6 as an independent and vital biomarker to detect COVID-19 disease severity.

### Lymphocyte count

Lymphocytopenia (ALC = <1000 cells/μl blood) is a common systemic manifestation of many immunodeficiencies and viral infections [80]. Previously, SARS-CoV and MERS-CoV had demonstrated lymphocytopenia as a vital feature of the viral infection [81]. A low lymphocyte count is rarely observed in COVID-19-infected children, but frequently observed in the elderly infected population [82].

A recent study reported that the majority of the COVID-19 patients had an abnormally low count of lymphocytes [83]. On the contrary, a higher CD4^+^ and CD8^+^ cell count was seen to contribute to a reduction in the severity of the disease [83]. Another study also reported that the average absolute lymphocyte (ALC) count was significantly lower in COVID-19 patients who needed ICU admission than in the patients who did not [84]. A systemic review and meta-analysis report compared the lymphocyte count of the severe patient group with the nonsevere group and concluded that lymphocytopenia is associated with a threefold increase in the risk of disease severity in SARS-CoV-2 infection (Table 2) [52,53,85].

Several factors may be responsible for the lymphocytopenia in severe COVID-19 patients, including inflammatory cytokine storm, direct viral attack on lymphocytes, increased level of pro-inflammatory cytokines, increased serum levels of IL-6 and interference with T-cell proliferation by downregulation of several gene expressions by the virus [82]. All these observations suggest that lymphocytopenia can be a vital biomarker to predict COVID-19 disease severity. An investigation regarding the underlying cause of lymphocytopenia and its reliability as a biomarker to predict disease severity should be further explored.

### Platelet count

Thrombocytopenia, a condition characterized by abnormally low levels of platelets, is a common effect of many viral infections. A study in a different cohort of COVID-19 patients reported that 36.2–55% of the patients had thrombocytopenia and the abnormalities were even worse in severe patients [42,86]. As a result of the reduced level of platelets, different cardiovascular events are anticipated such as venous and arterial thrombosis, of which there is already evidence in COVID-19 patients. These aggregated events can eventually lead to the multiorgan failure and death of the infected individual [87,88]. A meta-analysis showed thrombocytopenia is associated with a threefold increased risk of severe infection; platelet count was significantly lower in severe patients and even lower in patients who died of COVID-19 [89]. Another case report showed that the platelet count of a 57-year-old declined significantly during the initial stages of hospital admission, but increased in response to treatments during the latter stages [90]. These evidences suggest that platelet counts can be important biomarkers to detect disease progression and severity in SARS-CoV-2 infections.

Multiple factors may influence the abnormally reduced number of platelet in COVID-19 patients. It has been suggested that SARS-CoV-2 can enter the bone marrow and inhibit the process of hematopoiesis through certain receptors resulting in a decreased platelet count in patients [91]. Some studies suggest that the cytokine storm-generated during the COVID-19 infection may damage the hematopoietic progenitor stem cells in the bone marrow that reduces the primary production of platelets resulting in abnormal hematopoiesis and a low platelet count [92]. Enhanced immune response due to viral infection may also be a reason for the extensive destruction of platelets [91]. Moreover, damage to the lung tissues and endothelial cells caused by SARS-CoV-2 may trigger microthrombin formation and platelet accumulation, which may increase platelet consumption at the site of injury [54,55,86]. Since several reports suggesting a correlation between platelet count and disease severity in COVID-19 exists, further evidence should be collected to evaluate the importance of low platelet count as a biomarker of worse COVID-19 disease prognosis (Table 2).

### D-Dimer

D-Dimer, a fibrin degradation product, is an indicator of hypercoagulation state, secondary fibrinolysis, the abnormal process of coagulation and thrombosis [93–95]. D-Dimer assays are commonly used in clinical practices to indicate an increased risk of abnormal blood clotting and for the diagnosis of deep-vein thrombosis or pulmonary embolism. On analysis of patients with severe community-acquired pneumonia, it was found out that an increased D-Dimer level is related to a higher risk for complications and higher mortality rate [96,97].

Several reports have suggested an elevated concentration of D-Dimer is associated with COVID-19 severity. Studies reported that an elevated level of D-Dimer in severe COVID-19 patients compared with mild cases (Table 2) [98], but its level was significantly elevated in ICU patients compared with non-ICU patients [7]. Similarly, in a Chinese COVID-19 cohort showed that D-Dimer levels >2.0 mg/l during hospital admission were the only variable that could be linked to the increased risk of mortality [99].

D-Dimer may serve as an indicator for severe viral infection as a viral infection is likely to develop into sepsis and induce coagulation dysfunction, which in turn can serve as a common representation of severe disease progression. Moreover, the cytokine storm generated during a COVID-19 infection can recruit several inflammatory and proinflammatory mediators to create an imbalance in the coagulation process and fibrinolysis, ultimately elevating the level of D-Dimer in patients [100]. In a cohort of hospitalized COVID-19 patients, it was reported that anticoagulant treatment could reduce the risk of mortality, especially in patients receiving mechanical ventilation [56,57,100]. Since evidence suggesting a correlation of elevated D-Dimer levels to COVID-19 infection severity has been found, it can be used as an efficient prognostic biomarker for predicting worse disease outcome and also be targeted to reduce mortality in severe patients.

### Lactate dehydrogenase

Lactate dehydrogenase (LDH) is an intracellular glycolytic enzyme implicated in the conversion of lactate to pyruvate and is present in higher amounts in almost all the body cells. It is considered as an inflammatory marker and may serve as a common indicator of acute or severe tissue damage [101]. It has also been considered to be the biomarker of cardiovascular event like myocardial ischemia and a recent study suggests an association with coronary artery disease [102]. Evidence also suggests that serum LDH levels can serve as a nonspecific indicator of cellular death and is associated with poor prognosis in malignancy [103,104]. LDH was considered to be one of the biomarkers strongly associated with ARDS mortality [105,106]. Elevated LDH levels were also found in patients with SARS-CoV and MERS-CoV infection [107].

Elevated serum LDH levels have been attributed to increased disease severity and a higher risk of in-hospital COVID-19 mortality. A recent pooled analysis showed a >sixfold increase in odds of developing severe COVID-19 infection outcomes and a >16-fold increase in odds of mortality in patients with high levels of LDH (Table 2) [108–110]. A positive correlation of increased LDH levels with the CRP and cardiac troponin I (cTnI) level and a negative correlation with lymphocyte count in severe COVID-19 patients were also reported [109].

Cytokine-mediated lung tissue damage, which is a primary feature of severe SARS-CoV-2 infection, may be responsible for the elevated release of LDH [111]. The greater amounts of LDH released in circulation as the severe form of interstitial pneumonia may evolve into ARDS, which is one major hallmark of COVID-19 infection [108]. Moreover, normalizing the LDH titer has been successful in predicting treatment success in several cases and should be further investigated [58,112].

### Cardiac troponin

Cardiac troponins are a group of cardiac regulatory proteins that control muscular contraction and may serve as an indicator to detect heart muscle injury. Cardiac troponin is still the only standard biomarker for the detection of acute myocardial infarction. The onset of myocardial infarction is demonstrated by an elevated level of cardiac troponin [113]. However, this protein is often associated with viral infection-induced sepsis and the incidence of acute respiratory infections as a marker of disease severity [114–116]. Previous observations regarding the SARS-CoV and MERS-CoV outbreaks provided enough evidence of myocardial injury and myocarditis [117].

Cardiac troponin I (cTnI) was found to be marginally increased in the mild severe patients and significantly higher in severe COVID-19 patients [118]. A considerably high cTnI level was also reported in COVID-19 patients suffered from cardiac injury [119]. Furthermore, one out of five hospitalized COVID-19 patients were reported to have significantly elevated levels of cardiac troponin and these patients were more likely to require invasive or noninvasive ventilation [120]. The mortality rate in patients with myocardial injury was found to be tenfold enhanced compared to patients without myocardial injury [120].

Several mechanisms may explain this phenomenon of elevated cardiac troponin in severe COVID-19 patients. ACE-2 is highly expressed in the pericytes of the human heart tissues, which allows SARS-CoV-2 to directly enter the heart cells and cause severe damage [121]. The virus can induce direct virus-mediated lysis of cardiomyocytes and ventricular dysfunction by inducing a strong T-cell response [122,123]. SARS-CoV-2-induced myocarditis has been reported to be responsible for up to 7% of COVID-19-related deaths [124]. Early measurement of cardiac troponin levels could facilitate the understanding of the systemic consequence of the disease and interpret the requirement of inotropes, vasopressors, diuretics and other additional therapeutic implications in patients with cardiac dysfunction [59,60,125]. All these shreds of evidence suggest that cardiac troponin levels can be a vital biomarker in predicting COVID-19 severity, especially in people with a history of cardiovascular disease, however, further investigation to understand the underlying mechanisms should be undertaken in order to ascertain its validity as a biomarker for detecting severe disease outcomes (Table 2).

### Serum ferritin

Ferritin, an iron-storing protein, is a key mediator of immune dysfunction. Serum ferritin is an indicator of inflammation, released by the damaged cells, but whether the presence of this protein in serum is usual or not has yet to be ascertained [126]. Researchers found increased levels of serum ferritin in different lung diseases such as idiopathic pulmonary fibrosis [127], and reflecting its potential elevated release from damaged lung cells after infection with respiratory viruses. Increased level of serum ferritin has been reported in disease severity as well as fatal outcomes in COVID-19 patients (Table 2) [128–131]. Although hyper-ferritinemia was observed in severe cases of COVID-19, the concentration of ferritin was within the normal range for the nonsevere disease cases [132].

Higher ferritin levels in COVID-19 are not exclusively driven by SARS-CoV-2 infection; it may also occur due to other inflammatory diseases or several inflammatory cytokines such as IL-6 [133]. Moreover, some COVID-19 patients may have higher ferritin levels due to the pre-existence of comorbid conditions such as bacterial infections, autoimmune diseases, pre-existing neoplasm [61,62,134]. Further exploration of the reliability of serum ferritin levels as a biomarker for the prediction of COVID-19 disease prognosis should be undertaken.

### Acute kidney injury

Although the SARS-CoV-2 primarily damages the lung tissues and generates symptoms of acute respiratory failure, it has been found to enter the bloodstream and accumulate in several other organs causing secondary organ damage [135]. Among the secondary organs, the incidence and clinical characteristics of acute kidney injury (AKI) have been reported in many laboratory-confirmed COVID-19 patients [136]. A recent study conducted on 116 patients found an incidence of AKI in 18.1% of the patients and the majority were in critical condition, as well as had a higher prevalence of combined shock. Whereas, among the non-AKI patients the incidence of combined shock was significantly lower, and only a quarter of the patients were critical [136]. A similar phenomenon was also observed during the SARS-CoV outbreak, where patients with acute renal impairment in addition to the viral infection had a higher mortality rate in contrast to patients affected by the virus, but no renal impairment [137].

Various degrees of renal dysfunction have been reported in COVID-19 patients, characterized by increased levels of blood urea nitrogen (BUN) and serum creatinine [138]. A study reported that 27% of the COVID-19 patients showed elevated BUN levels, and of those, two-thirds of the patients with elevated BUN levels and serum creatinine levels over 200 μmol/l had died [139]. Another study also reported that more than 40% of the hospitalized patients had evidence of kidney disease and were associated with higher cases of in-hospital mortality [135].

One possible explanation of the high incidence of AKI in COVID-19 patients may be related to the mechanism of SARS-CoV-2 entrance in cells. Renal tubular epithelial cells have a high expression of ACE-2 protein, the receptor that binds to and facilitates viral entrance in cells [140]. The higher concentration of inflammatory mediators in response to a cytokine storm can also result in renal impairment [141]. Another possible explanation can be the high prevalence and history of chronic kidney diseases in COVID-19 patients at the time of hospital admission [142]. Dysfunction of the endothelial system, coagulopathy and activation of the complement pathway may also contribute to the development of AKI in a subset of COVID-19 patients [143]. Since AKI has been strongly associated with increased COVID-19 mortality and morbidity, it can serve as an important biomarker for the prediction of disease severity and as a potential therapeutic target to reduce COVID-19-related deaths.

### Others

Besides these, a number of other biomarkers can be considered for the diagnosis of COVID-19. Different types of viral protein-coding genes can be used in the appropriate diagnosis of COVID-19 patients. In fact, the most commonly used reverse transcriptase polymerase chain reaction (RT-PCR) technology utilizes the polymerization reaction of different genes of SARS-CoV-2 involved in the expression of structural and nonstructural proteins. These genes are predominantly involved in the expression of spike protein (S), membrane protein (M), nucleocapsid protein (N), RNA-dependent RNA polymerase (RdRp) and so on. Use of nasopharyngeal or oropharyngeal swab of infected patients is enough to extract and amplify the above-mentioned viral genes when using RT-PCR technology for COVID-19 diagnosis [144,145]. Loss of smell and taste have been characterized as a potential early symptom of COVID-19 infection [146]. In a cohort of Iranian hospitalized COVID-19 patients, >98% of patients had an impaired sense of smell characterized by psychophysical olfactory testing [147]. It is suggested that SARS-CoV-2 targets the olfactory and gustatory system and may be involved in the peripheral damage of the olfactory receptor cells in the nasal neuroepithelium [148]. Intra-nasal administration of SARS-CoV-2 in a transgenic mice model has demonstrated that the virus can penetrate the brain through the olfactory bulb [149]. Since the high prevalence of smell dysfunction has been reported in several cases, it can serve as a vital biomarker for COVID-19 screening.

Vitamin D is an important regulator of host inflammatory and immune response. Recent studies showed that a decreased level of 25 hydroxyvitamin D or 25(OH)D has been associated with the severity of COVID-19. It has been reported that hospitalized COVID-19 patients with vitamin D deficiency experienced a higher risk of developing ARDS compared with patients with normal vitamin D levels [150]. Healthy vitamin D levels can turn down the cytokine storm generated by the virus and reduce the overproduction of pro-inflammatory cytokines [150]. However, in a recent MR study, the results indicated no linear causal relationship of 25(OH)D concentration with coronavirus disease severity and susceptibility [151]. Further investigation paying more attention to the randomization control trials must be done to better evaluate the association between vitamin D levels and COVID-19 disease prognosis.

Serum IgA, IgG and IgM immunoglobulins that are produced by the host immune system in response to SARS-CoV-2 infection could serve as a potential biomarker in COVID-19 diagnosis. A recent study demonstrated that IgA levels reached the peak value during the initial 16–20 days after disease onset and then began to decline but continues to remain at relatively high value for a considerable period, IgG level was initially low but started to rise at 15 days after disease onset and stayed at a relatively high value for a considerable period, and IgM levels peaked at early stage, but the reading was comparatively lower than IgA and IgG [152]. Overall, IgM and IgG levels were found to be elevated in both moderate and severe COVID-19 patients compared with the mild cases, whereas IgA levels were only found to be elevated in severe cases of the infection [152]. Another study also showed increased serum IgG and IgM antibodies in severe COVID-19 patients compared with mild cases [153]. These shreds of evidence suggest that serological anti-SARS-CoV-2 antibody tests can serve as a potential biomarker to predict disease severity.

Some epidemiological studies suggest that one of the major predictors of COVID-19 severity is age, pre-existing disease conditions and habits associated with an unhealthy lifestyle [154]. So, biological age is more reliable in predicting worse COVID-19 prognosis than chronological age [154]. Glycan clock is one of the biomarkers that can be used for the prediction of biological age and can predict the prevalence and susceptibility of COVID-19 infection [155]. Besides, glycan also serves as a principal regulator of host immune responses [155]. Based on such findings, it is suggested that biological age may serve as an important biomarker in COVID-19 severity.

## Conclusion

Due to the contagious nature of the virus and lack of any specific approved treatment, it becomes important to ensure early stratification of the infected patients to reduce the risk of transmission among susceptible groups as well as to allocate optimal resources to the patients who are in severe condition. Biomarkers can play a significant role by facilitating timely diagnosis, risk monitoring and directing the optimal treatment of COVID-19 patients. Based on the aforementioned promising evidences, it is apparent that biomarkers are essential to optimize the COVID-19 prognosis and hereby to minimize the mortality rates. However, individual variation among patients in biomarker levels may interrupt the optimal interpretation of disease prognosis. Therefore, further studies must be conducted to evaluate and identify best potential biomarker for COVID-19 diagnosis.

## Future perspective

The severe global crisis generated by the highly contagious SARS-CoV-2 has affected over 103 million people and has resulted in around 2.24 million deaths worldwide. The spread of the virus is so swift that the general population, healthcare professionals and regulatory authorities from different countries have employed vastly different, but effective strategies to battle against the virus. In this situation, early diagnosis and effective management of the infected population are critical. Biomarkers can play a key role in this regard by ensuring the efficient diagnosis as well as guiding improvements in the clinical management of patients with COVID-19. More significantly, the use of biomarkers can aid the development and approval of biological agents and therapeutics in addition to evaluating COVID-19 pathogenesis and accessing disease prognosis. Although research is still in its early stages, the characteristic features of different biomarkers during the disease progression could help categorizing and understanding the underlying disease pathomechanism. Moreover, the identification of efficient biomarkers can also assist in predicting future complications or severity of the disease. In addition, the identification of integral biomarkers of the SARS-CoV-2 may facilitate a much more robust response against future respiratory viral outbreaks with the potential of creating global pandemics. The accumulated knowledge regarding SARS-CoV-2 infection biomarkers might be utilized to generate a much more efficient resource allocation in tackling future outbreaks and pandemics and more research works are warranted in this regard.

**Executive summary**

- Biomarkers can play a significant role in the early diagnosis and differentiation of SARS-CoV-2 infection from other pulmonary infections exhibiting almost similar symptoms.
- SARS-CoV-2 invades a human host cell through five significant steps – attachment, penetration, biosynthesis, maturation and release.
- Clinical presentation of chronic obstructive pulmonary disease (COPD) and SARS-CoV-2 infected patients are quite similar which makes it difficult to distinguish the symptoms. Biomarkers have proven to be effective in differentiating between these two classes of patients. The most determining biomarkers between these two groups are BASP, PTA, PT and INR. Some of the coagulation parameters were different between these two groups as well.
- PCT values were expected to remain within the minimum range in patients with noncomplicated COVID-19 infection as the increased concentration of IFN-γ can inhibit PCT production. Nevertheless, PCT levels were reported to be increased among severe COVID-19 patients, which may be attributed to viral-induced lung tissue damage and bacterial translocation, resulting in bacterial coinfection.
- Under normal conditions, the concentration of CRP in the blood is less than 10 mg/l, but the concentration may increase up to 1000-fold, serving as a significant indicator of COVID-19 infection severity.
- SAA levels are significantly increased in severe COVID-19 patients. Studies suggest that SAA can be used as an independent predictive biomarker of COVID-19. The expression level is significantly higher in acute phase infection compared with the convalescent phase infection.
- IL-6 levels are around three-times higher in the severe COVID-19 patients than the nonsevere patients and can also be linked to higher mortality rates.
- Lymphocytopenia is associated with a threefold increased risk of disease severity in SARS-CoV-2 infection. The average ALC count is reported to be significantly lower in COVID-19 patients requiring ICU admission compared with patients who do not require ICU admission.
- Thrombocytopenia can be linked to different cardiovascular events like venous and arterial thrombosis that are highly prevalent as comorbid conditions in COVID-19 patients. Besides, the lower levels of platelets and associated events can eventually lead to multiorgan failure and death of the infected individual.
- Elevated D-Dimer levels might serve as an indicator for severe COVID-19 infection as the cytokine storm-generated during the viral pathogenesis can recruit several inflammatory mediators to create an imbalance of the coagulation process and fibrinolysis resulting in enhanced D-Dimer secretion.
- A recent pooled analysis showed a >sixfold increase in odds of developing severe COVID-19 infection outcomes and a >16-fold increase in odds of mortality in patients with elevated levels of LDH suggesting LDH as an effective biomarker to mark disease severity. Besides, this biomarker can also be used as a predictor of comorbid cardiovascular events in COVID-19 patients.
- High cardiac troponin levels are reported in COVID-19 patients, especially those suffering from cardiac injury or having previous history of cardiovascular diseases.
- Apart from these, major biomarkers like serum ferritin, AKI indicators, serum immunoglobulins, vitamin D levels, biological age, and loss of smell and taste can also be considered as crucial biomarkers in detecting and accessing COVID-19 disease severity.

## References

[1] Coronavirus disease (COVID-19) – World Health Organization. (2020). https://www.who.int/emergencies/diseases/novel-coronavirus-2019

[2] Coronavirus update (live): 46,495,184 cases and 1,201,924 deaths from COVID-19 virus pandemic – Worldometer (2020). https://www.worldometers.info/coronavirus/

[3] Chan J, Kok K, Zhu Z Genomic characterization of the 2019 novel human-pathogenic coronavirus isolated from a patient with atypical pneumonia after visiting Wuhan. Emerg. Microbes Infect. 9(1), 221–236 (2020). 31987001 10.1080/22221751.2020.1719902 PMC7067204

[4] Hoffmann M, Kleine-Weber H, Schroeder S SARS-CoV-2 cell entry depends on ACE2 and TMPRSS2 and is blocked by a clinically proven protease inhibitor. Cell 181(2), 271–280 (2020). 32142651 10.1016/j.cell.2020.02.052 PMC7102627

[5] Islam H, Rahman A, Masud J A generalized overview of SARS-CoV-2: where does the current knowledge stand? Electronic J. Gen. Med. 17(6), em251 (2020).

[6] Ponti G, Maccaferri M, Ruini C, Tomasi A, Ozben T. Biomarkers associated with COVID-19 disease progression. Crit. Rev. Clin. Lab. Sci. 57(6), 389–399 (2020). 32503382 10.1080/10408363.2020.1770685 PMC7284147

[7] Huang C, Wang Y, Li X Clinical features of patients infected with 2019 novel coronavirus in Wuhan, China. Lancet 395(10223), 497–506 (2020). 31986264 10.1016/S0140-6736(20)30183-5 PMC7159299

[8] Zaim S, Chong J, Sankaranarayanan V, Harky A. COVID-19 and multiorgan response. Curr. Probl. Cardiol. 45(8), 100618 (2020). 32439197 10.1016/j.cpcardiol.2020.100618 PMC7187881

[9] Pierce J, McCabe S, White N, Clancy R. Biomarkers. Am. J. Nursing 112(9), 52–58 (2012). 10.1097/01.NAJ.0000418926.83718.28 22932054

[10] Kermali M, Khalsa R, Pillai K, Ismail Z, Harky A. The role of biomarkers in diagnosis of COVID-19 – a systematic review. Life Sci. 254, 117788 (2020). 32475810 10.1016/j.lfs.2020.117788 PMC7219356

[11] Zhang L, Guo H. Biomarkers of COVID-19 and technologies to combat SARS-CoV-2. Adv. Biomark. Sci. Technol. 2, 1–23 (2020). 33511330 10.1016/j.abst.2020.08.001 PMC7435336

[12] Malik P, Patel U, Mehta D Biomarkers and outcomes of COVID-19 hospitalisations: systematic review and meta-analysis. BMJ Evid. Based Med. 26(3), 108–107 (2020). 10.1136/bmjebm-2020-111536 PMC7493072 32934000

[13] Pneumonia Symptoms. WebMD (2020). https://www.webmd.com/lung/understanding-pneumonia-symptoms

[14] Dai J, Du Y, Gao J Difference in biomarkers between COVID-19 patients and other pulmonary infection patients. Infect. Drug Resist. 13, 2609–2615 (2020). 32801798 10.2147/IDR.S257936 PMC7397211

[15] Bosch B, van der Zee R, de Haan C, Rottier P. The coronavirus spike protein is a class I virus fusion protein: structural and functional characterization of the fusion core complex. J. Virol. 77(16), 8801–8811 (2003). 12885899 10.1128/JVI.77.16.8801-8811.2003 PMC167208

[16] V'kovski P, Kratzel A, Steiner S, Stalder H, Thiel V. Coronavirus biology and replication: implications for SARS-CoV-2. Nat. Rev. Microbiol. 18(10), 1–6 (2020). 33116300 10.1038/s41579-020-00468-6 PMC7592455

[17] Guruprasad L. Human SARS CoV-2 spike protein mutations. Proteins 2021, 1–8 (2021). 10.1002/prot.26042 PMC8014176 33423311

[18] Why does the coronavirus spread so easily between people? (2020). https://www.nature.com/articles/d41586020-00660-x 10.1038/d41586-020-00660-x 32157230

[19] Li G, Fan Y, Lai Y Coronavirus infections, and immune responses. J. Med. Virol. 92(4), 424–432 (2020). 31981224 10.1002/jmv.25685 PMC7166547

[20] Iqbal HMN, Romero-Castillo K, Bilal M, Parra-Saldivar R. The emergence of novel-coronavirus and its replication cycle – an overview. J. Pure Appl. Microbiol. 14(1), 16 (2020).

[21] Tripp RA, Tompkins SM (). Roles of Host Gene and Non-Coding RNA Expression in Virus Infection. Springer International Publishing, Switzerland (2018).

[22] Qin C, Zhou L, Hu Z Dysregulation of immune response in patients with coronavirus 2019 (COVID-19) in Wuhan, China. Clin. Infect. Dis. 71(15), 762–768 (2020). 32161940 10.1093/cid/ciaa248 PMC7108125

[23] Ragab D, Salah Eldin H, Taeimah M, Khattab R, Salem R. The COVID-19 cytokine storm; what we know so far. Front. Immunol. 11, 1446 (2020). 32612617 10.3389/fimmu.2020.01446 PMC7308649

[24] Busse W, Corren J, Lanier B, McAlary M, Fowler-Taylor A, Cioppa G Omalizumab, an anti-IgE recombinant humanized monoclonal antibody, for the treatment of severe allergies asthma. J. Allergy Clin. Immunol. 108(2), 184–190 (2001). 11496232 10.1067/mai.2001.117880

[25] Signs and Symptoms of COPD. WebMD (2020). https://www.webmd.com/lung/copd/what-are-symptoms-of-copd

[26] Chronic Bronchitis. WebMD (2020). https://www.webmd.com/lung/copd/copd-chronic-bronchitis

[27] Pneumonia Symptoms and Diagnosis. Lung.org (2020). https://www.lung.org/lung-health-diseases/lung-disease-lookup/pneumonia/symptoms-and-diagnosis#:∼:text=The%20symptoms%20of%20viral%20pneumonia,of%20breath%20and%20muscle%20pain

[28] Bronchitis – symptoms and causes. Mayo Clinic (2020). https://www.mayoclinic.org/diseases-conditions/bronchitis/symptoms-causes/syc-20355566

[29] COPD and Headaches: Causes, Symptoms, and Treatment. Healthline (2020). https://www.healthline.com/health/copd/headache#:∼:text=Headaches%20from%20COPD%20happen%20from,at%20risk%20for%20sleep%20apnea

[30] COVID-19: Use rapid tests to identify important biomarkers and predict the course of the disease. RealWire (2021). https://www.realwire.com/releases/Use-rapid-tests-to-identify-important-biomarkers

[31] Min B. Basophils: what they ‘can do’ versus what they ‘actually do’. Nat. Immunol. (12), 1333–9 (2008). 19008933 10.1038/ni.f.217

[32] O'Connell PA, Surette AP, Liwski RS, Svenningsson P, Waisman DM. S100A10 regulates plasminogen-dependent macrophage invasion. Blood 116(7), 1136–1146 (2010). 20424186 10.1182/blood-2010-01-264754

[33] Berri F, Rimmelzwaan GF, Hanss M Plasminogen controls inflammation and pathogenesis of influenza virus infections via fibrinolysis. PLoS Pathog. 9(3), e1003229 (2013). 23555246 10.1371/journal.ppat.1003229 PMC3605290

[34] Cervellin G, Pedrazzoni M, Passeri M. New polypeptide hormonal candidates encoded by the calcitonin gene. Recenti. Prog. Med. 74(9), 979–985 (1983). 6689214

[35] Marston HD, Dixon DM, Knisely JM, Palmore TN, Fauci AS. Antimicrobial resistance. JAMA 316(11), 1193–1204 (2016). 27654605 10.1001/jama.2016.11764

[36] Becker KL, Nylen ES, White JC, Muller B, Snider RH Jr. Procalcitonin and the calcitonin gene family of peptides in inflammation, infection, and sepsis: a journey from calcitonin back to its precursors. J. Clin. Endocrinol. Metab. 89(4), 1512–1525 (2004). 15070906 10.1210/jc.2002-021444

[37] Schuetz P, Albrich W, Mueller B. Procalcitonin for diagnosis of infection and guide to antibiotic decisions: past, present and future. BMC Med. 9(1), 1–9 (2011). 21936959 10.1186/1741-7015-9-107 PMC3186747

[38] Lippi G, Plebani M. Procalcitonin in patients with severe coronavirus disease 2019 (COVID-19): a meta-analysis. Clin. Chim. Acta 505, 190–191 (2020). 32145275 10.1016/j.cca.2020.03.004 PMC7094472

[39] Health Management. The role of procalcitonin for risk assessment and treatment of COVID-19 patients (2020). https://healthmanagement.org/c/healthmanagement/issuearticle/the-role-of-procalcitonin-for-risk-assessment-and-treatment-of-covid-19-patients

[40] Wang D, Hu B, Hu C Clinical characteristics of 138 hospitalized patients with 2019 novel coronavirus–infected pneumonia in Wuhan, China. JAMA 323(11), 1061–1069 (2020). 32031570 10.1001/jama.2020.1585 PMC7042881

[41] Zhang JJ, Dong X, Cao YY Clinical characteristics of 140 patients infected with SARS-CoV-2 in Wuhan, China. Allergy 75(7), 1730–1741 (2020). 32077115 10.1111/all.14238

[42] Guan WJ, Ni ZY, Hu Y Clinical characteristics of coronavirus disease 2019 in China. N. Engl. J. Med. 382(18), 1708–1720 (2020). 32109013 10.1056/NEJMoa2002032 PMC7092819

[43] Krause M, Douin DJ, Tran TT, Fernandez-Bustamante A, Aftab M, Bartels K. Association between procalcitonin levels and duration of mechanical ventilation in COVID-19 patients. PLoS ONE 15(9), e0239174 (2020). 32946466 10.1371/journal.pone.0239174 PMC7500634

[44] Procalcitonin (PCT): reference range of procalcitonin, interpretation of procalcitonin levels, collection and panels (2020). https://emedicine.medscape.com/article/2096589-overview#:∼:text=The%20reference%20value%20of%20PCT,below%20the%20level%20of%20detection

[45] Hu R, Han C, Pei S, Yin M, Chen X. Procalcitonin levels in COVID-19 patients. Int. J. Antimicrob. Agents 56(2), 106051 (2020). 32534186 10.1016/j.ijantimicag.2020.106051 PMC7286278

[46] C-reactive protein test – Mayo Clinic. Mayoclinic.org (2020). https://www.mayoclinic.org/tests-procedures/c-reactive-protein-test/about/pac-20385228#:∼:text=For%20a%20standard%20CRP%20test,testing%20to%20determine%20the%20cause

[47] Kermali M, Khalsa R, Pillai K, Ismail Z, Harky A. The role of biomarkers in diagnosis of COVID-19 – a systematic review. Life Sci. 254, 117788 (2020). 32475810 10.1016/j.lfs.2020.117788 PMC7219356

[48] Targońska-Stępniak B, Majdan M. Serum amyloid A as a marker of persistent inflammation and an indicator of cardiovascular and renal involvement in patients with rheumatoid arthritis. Mediators Inflamm. 2014, 1–7 (2014). 10.1155/2014/793628 PMC4265690 25525305

[49] Fu J, Huang PP, Zhang S The value of serum amyloid A for predicting the severity and recovery of COVID-19. Exp. Ther. Med. 20(4), 3571–3577 (2020). 32855710 10.3892/etm.2020.9114 PMC7444421

[50] Khan A, Ali Z. Normal ranges for acute phase reactants (interleukin-6, tumour necrosis factor-alpha and C-reactive protein) in umbilical cord blood of healthy term neonates at the Mount Hope Women's Hospital, Trinidad. West Indian Med. J. 63(5), 465 (2014). 25781284 10.7727/wimj.2012.133 PMC4655679

[51] Herold T, Jurinovic V, Arnreich C Level of IL-6 predicts respiratory failure in hospitalized symptomatic COVID-19 patients. MedRxiv (2020). https://www.medrxiv.org/content/10.1101/2020.04.01.20047381v2

[52] Lymphocytes: levels, ranges, and functions (2020). https://www.medicalnewstoday.com/articles/320987#normal-ranges-and-levels

[53] Wagner J, DuPont A, Larson S, Cash B, Farooq A. Absolute lymphocyte count is a prognostic marker in Covid-19: a retrospective cohort review. Int. J. Lab. Hematol. 42(6), 761–765 (2020). 32779838 10.1111/ijlh.13288 PMC7405282

[54] Chen N, Zhou M, Dong X Epidemiological and clinical characteristics of 99 cases of 2019 novel coronavirus pneumonia in Wuhan, China: a descriptive study. Lancet 395(10223), 507–513 (2020). 32007143 10.1016/S0140-6736(20)30211-7 PMC7135076

[55] Huang C, Wang Y, Li X Clinical features of patients infected with 2019 novel coronavirus in Wuhan, China. Lancet 395(10223), 497–506 (2020). 31986264 10.1016/S0140-6736(20)30183-5 PMC7159299

[56] Emedicine.medscape.com. D-Dimer: reference range, interpretation, collection and panels (2020). https://emedicine.medscape.com/article/2085111-overview

[57] Vidali S, Morosetti D, Cossu E D-dimer as an indicator of prognosis in SARS-CoV-2 infection: a systematic review. ERJ Open Res. 6, 2 (2020). 10.1183/23120541.00260-2020 PMC7357271 32685436

[58] Michigan Medicine. Lactic acid dehydrogenase (LDH) (2020). https://www.uofmhealth.org/health-library/tv6985#:∼:text=Results,L%20to%204.68%20microkatals%2FL

[59] Normal troponin levels: what high levels mean, plus causes (2020). https://www.medicalnewstoday.com/articles/325415

[60] SOLACI. Myocardial injury in one third of COVID-19 patients (2020). https://solaci.org/en/2020/07/01/myocardial-injury-in-one-third-of-covid-19-patients/

[61] Ferritin blood test: understand results of high, low, and normal levels (2020). https://www.medicinenet.com/ferritin_blood_test/article.htm

[62] Vargas-Vargas M, Cortés-Rojo C. Ferritin levels and COVID-19. Revista Panamericana de Salud Pública 44, 1 (2020). 10.26633/RPSP.2020.72 PMC7286435 32547616

[63] Wang G, Wu C, Zhang Q C-reactive protein level may predict the risk of COVID-19 aggravation. Open Forum Infect. Dis. 7(5), ofaa153 (2020). 32455147 10.1093/ofid/ofaa153 PMC7197542

[64] Young B, Gleeson M, Cripps AW. C-reactive protein: a critical review. Pathology 23(2), 118–124 (1991). 1720888 10.3109/00313029109060809

[65] Thompson D, Pepys MB, Wood SP. The physiological structure of human C-reactive protein and its complex with phosphocholine. Structure 7(2), 169–177 (1999). 10368284 10.1016/S0969-2126(99)80023-9

[66] Ridker PM. Clinical application of C-reactive protein for cardiovascular disease detection and prevention. Circulation 107(3), 363–369 (2003). 12551853 10.1161/01.cir.0000053730.47739.3c

[67] Wang L. C-reactive protein levels in the early stage of COVID-19. Medecine et Maladies Infectieuses 50(4), 332–334 (2020). 32243911 10.1016/j.medmal.2020.03.007 PMC7146693

[68] Ali N. Elevated level of C-reactive protein may be an early marker to predict risk for severity of COVID-19. J. Med. Virol. 92(11), 2409–2411 (2020). 32516845 10.1002/jmv.26097 PMC7301027

[69] Lin HY, Tan GQ, Liu Y, Lin SQ. The prognostic value of serum amyloid A in solid tumors: a meta-analysis. Cancer Cell Int. 19(1), 1–0 (2019). 10.1186/s12935-019-0783-4 PMC6425599 30930691

[70] Mo XN, Su ZQ, Lei CL Serum amyloid A is a predictor for prognosis of COVID-19. Respirology 7(65), 764–725 (2020). 10.1111/resp.13840 PMC7272841 32406576

[71] Li H, Xiang X, Ren H Serum Amyloid A is a biomarker of severe Coronavirus Disease and poor prognosis. J. Infect. 80(6), 646–655 (2020). 32277967 10.1016/j.jinf.2020.03.035 PMC7141628

[72] Zhang Y, Wang D, Lin M Serum amyloid A protein as a potential biomarker useful in monitoring the course of COVID-19: a retrospectively studied. Research Square https://www.researchsquare.com/article/rs-19724/v1 (2020).

[73] Nakayama T, Sonoda S, Urano T, Yamada T, Okada M. Monitoring both serum amyloid protein A and C-reactive protein as inflammatory markers in infectious diseases. Clin. Chem. 39(2), 293–297 (1993). 8381732

[74] Tanaka T, Narazaki M, Kishimoto T. IL-6 in inflammation, immunity, and disease. Cold Spring Harb. Perspect. Bio. 6(10), a016295 (2014). 25190079 10.1101/cshperspect.a016295 PMC4176007

[75] Grifoni E, Valoriani A, Cei F Interleukin-6 as prognosticator in patients with COVID-19. J. Infect. 81(3), 452–482 (2020). 10.1016/j.jinf.2020.06.008 PMC7278637 32526326

[76] Herold T, Jurinovic V, Arnreich C Elevated levels of IL-6 and CRP predict the need for mechanical ventilation in COVID-19. J. Allergy Clin. Immunol. 146(1), 128–136 (2020). 32425269 10.1016/j.jaci.2020.05.008 PMC7233239

[77] Aziz M, Fatima R, Assaly R. Elevated interleukin-6 and severe COVID-19: a meta-analysis. J. Med. Virol. 92(11), 2283–2285 (2020). 32343429 10.1002/jmv.25948 PMC7267383

[78] Liu Z, Li J, Chen D Dynamic interleukin-6 level changes as a prognostic indicator in patients with COVID-19. Front. Pharmacol. 11, 1093 (2020). 32765283 10.3389/fphar.2020.01093 PMC7379481

[79] Campochiaro C, Dagna L. The conundrum of interleukin-6 blockade in COVID-19. Lancet Rheumatol. 2(10), e579–e580 (2020). 32838322 10.1016/S2665-9913(20)30287-3 PMC7428300

[80] Kasper D, Fauci A, Hauser S, Longo D, Jameson J, Loscalzo J. Harrison's Principles of Internal Medicine. McGraw-Hill, NY, USA (2015).

[81] Yang X, Yu Y, Xu J Clinical course and outcomes of critically ill patients with SARS-CoV-2 pneumonia in Wuhan, China: a single-centered, retrospective, observational study. Lancet Respir. Med. 8(5), 475–481 (2020). 32105632 10.1016/S2213-2600(20)30079-5 PMC7102538

[82] Tavakolpour S, Rakhshandehroo T, Wei EX, Rashidian M. Lymphopenia during the COVID-19 infection: what it shows and what can be learned. Immunol. Lett. 225, 31 (2020). 32569607 10.1016/j.imlet.2020.06.013 PMC7305732

[83] Liu Z, Long W, Tu M Lymphocyte subset (CD4+, CD8+) counts reflect the severity of infection and predict the clinical outcomes in patients with COVID-19. J. Infect. 81(2), 318–356 (2020). 10.1016/j.jinf.2020.03.054 PMC7151318 32283159

[84] Wagner J, DuPont A, Larson S, Cash B, Farooq A. Absolute lymphocyte count is a prognostic marker in Covid-19: a retrospective cohort review. Int. J. Lab. Hematol. 42(6), 761–765 (2020). 32779838 10.1111/ijlh.13288 PMC7405282

[85] Zhao Q, Meng M, Kumar R Lymphopenia is associated with severe coronavirus disease 2019 (COVID-19) infections: a systemic review and meta-analysis. Int. J. Infect. Dis. 96, 131–135 (2020). 32376308 10.1016/j.ijid.2020.04.086 PMC7196544

[86] Xiong X, Chua GT, Chi S A comparison between chinese children infected with coronavirus disease-2019 and with severe acute respiratory syndrome 2003. J. Pediatrics 224, 30–36 (2020). 10.1016/j.jpeds.2020.06.041 PMC7301144 32565097

[87] Barrett TJ, Lee AH, Xia Y Platelet and vascular biomarkers associate with thrombosis and death in coronavirus disease. Circ. Res. 127(7), 945–947 (2020). 32757722 10.1161/CIRCRESAHA.120.317803 PMC7478197

[88] Salamanna F, Maglio M, Landini MP, Fini M. Platelet functions and activities as potential hematologic parameters related to Coronavirus Disease 2019 (Covid-19). Platelets 31(5), 627–632 (2020). 32397915 10.1080/09537104.2020.1762852

[89] Lippi G, Plebani M, Henry BM. Thrombocytopenia is associated with severe coronavirus disease 2019 (COVID-19) infections: a meta-analysis. Clin. Chim. Acta 506, 145–148 (2020). 32178975 10.1016/j.cca.2020.03.022 PMC7102663

[90] Sadr S, SeyedAlinaghi S, Ghiasvand F Isolated severe thrombocytopenia in a patient with COVID-19: a case report. IDCases 21, e00820 (2020). 32483524 10.1016/j.idcr.2020.e00820 PMC7255984

[91] Xu P, Zhou Q, Xu J. Mechanism of thrombocytopenia in COVID-19 patients. Ann. Hematol. 99(6), 1205–1208 (2020). 32296910 10.1007/s00277-020-04019-0 PMC7156897

[92] Jolicoeur P, Lamontagne L. Impairment of bone marrow pre-B and B cells in MHV3 chronically-infected mice. In: Corona and Related Viruses 193–195 (1995). 10.1007/978-1-4615-1899-0_33 8830480

[93] Giannitsis E, Mair J, Christersson C How to use D-dimer in acute cardiovascular care. Eur. Heart J. Acute Cardiovasc. Care 6(1), 69–80 (2017). 26450781 10.1177/2048872615610870

[94] Behrens K, Alexander WS. Cytokine control of megakaryopoiesis. Growth Factors 36(3–4), 89–103 (2018). 30318940 10.1080/08977194.2018.1498487

[95] Liu Y, Gayle AA, Wilder-Smith A, Rocklöv J. The reproductive number of COVID-19 is higher compared to SARS coronavirus. J. Travel Med. 27(2), 1–4 (2020). 10.1093/jtm/taaa021 PMC7074654 32052846

[96] Querol-Ribelles JM, Tenias JM, Grau E Plasma d-dimer levels correlate with outcomes in patients with community-acquired pneumonia. Chest 126(4), 1087–1092 (2004). 15486368 10.1378/chest.126.4.1087

[97] Snijders D, Schoorl M, Schoorl M, Bartels PC, van der Werf TS, Boersma WG. D-dimer levels in assessing severity and clinical outcome in patients with community-acquired pneumonia. A secondary analysis of a randomised clinical trial. Eur. J. Intern. Med. 23(5), 436–441 (2012). 22726372 10.1016/j.ejim.2011.10.019

[98] Wan S, Xiang YI, Fang W Clinical features and treatment of COVID-19 patients in northeast Chongqing. J. Med. Virol. 92(7), 797–806 (2020). 32198776 10.1002/jmv.25783 PMC7228368

[99] Yao Y, Cao J, Wang Q D-dimer as a biomarker for disease severity and mortality in COVID-19 patients: a case control study. J. Intens. Care 8(1), 1–1 (2020). 10.1186/s40560-020-00466-z PMC7348129 32665858

[100] Yu HH, Qin C, Chen M, Wang W, Tian DS. D-dimer level is associated with the severity of COVID-19. Thromb. Res. 195, 219–225 (2020). 32777639 10.1016/j.thromres.2020.07.047 PMC7384402

[101] Sepulveda JL.Challenges in routine clinical chemistry analysis: proteins and enzymes. Accurate Results Clin. Lab. 2, 141–163 (2019).

[102] Kopel E, Kivity S, Morag-Koren N, Segev S, Sidi Y. Relation of serum lactate dehydrogenase to coronary artery disease. Am. J. Cardiol. 110(12), 1717–1722 (2012). 22981267 10.1016/j.amjcard.2012.08.005

[103] Wimazal F, Sperr WR, Kundi M Prognostic significance of serial determinations of lactate dehydrogenase (LDH) in the follow-up of patients with myelodysplastic syndromes. Ann. Oncol. 19(5), 970–976 (2005). 10.1093/annonc/mdm595 18272915

[104] Scartozzi M, Giampieri R, Maccaroni E Pre-treatment lactate dehydrogenase levels as predictor of efficacy of first-line bevacizumab-based therapy in metastatic colorectal cancer patients. Br. J. Cancer 106(5), 799–804 (2012). 22315053 10.1038/bjc.2012.17 PMC3305976

[105] Terpstra ML, Aman J, van Nieuw Amerongen GP, Groeneveld AJ. Plasma biomarkers for acute respiratory distress syndrome: a systematic review and meta-analysis. Crit. Care Med. 42(3), 691–700 (2014). 24158164 10.1097/01.ccm.0000435669.60811.24

[106] Hoeboer SH, Oudemans-van Straaten HM, Groeneveld AJ. Albumin rather than C-reactive protein may be valuable in predicting and monitoring the severity and course of acute respiratory distress syndrome in critically ill patients with or at risk for the syndrome after new onset fever. BMC Pulm. Med. 15(1), 1–3 (2015). 25888398 10.1186/s12890-015-0015-1 PMC4381515

[107] Assiri A, Al-Tawfiq JA, Al-Rabeeah AA Epidemiological, demographic, and clinical characteristics of 47 cases of Middle East respiratory syndrome coronavirus disease from Saudi Arabia: a descriptive study. Lancet Infect. Dis. 13(9), 752–761 (2020). 10.1016/S1473-3099(13)70204-4 PMC7185445 23891402

[108] Henry BM, Aggarwal G, Wong J Lactate dehydrogenase levels predict coronavirus disease 2019 (COVID-19) severity and mortality: a pooled analysis. Am. J. Emerg. Med. 38(9), 1722–1726 (2020). 32738466 10.1016/j.ajem.2020.05.073 PMC7251362

[109] Han Y, Zhang H, Mu S Lactate dehydrogenase, an independent risk factor of severe COVID-19 patients: a retrospective and observational study. Aging 12(12), 11245 (2020). 32633729 10.18632/aging.103372 PMC7343511

[110] Dong X, Sun L, Li Y. Prognostic value of lactate dehydrogenase for in-hospital mortality in severe and critically ill patients with COVID-19. Int. J. Med. Sci. 17(14), 2225 (2020). 32922185 10.7150/ijms.47604 PMC7484664

[111] Martinez-Outschoorn UE, Prisco M, Ertel A Ketones and lactate increase cancer cell “stemness,” driving recurrence, metastasis and poor clinical outcome in breast cancer: achieving personalized medicine via Metabolo-Genomics. Cell Cycle 10(8), 1271–1286 (2011). 21512313 10.4161/cc.10.8.15330 PMC3117136

[112] Wu MY, Yao L, Wang Y Clinical evaluation of potential usefulness of serum lactate dehydrogenase (LDH) in 2019 novel coronavirus (COVID-19) pneumonia. Respir. Res. 21(1), 1–6 (2020). 32631317 10.1186/s12931-020-01427-8 PMC7336103

[113] Hochholzer W, Morrow DA, Giugliano RP. Novel biomarkers in cardiovascular disease: update 2010. Am. Heart J. 160(4), 583–594 (2010). 20934551 10.1016/j.ahj.2010.06.010

[114] Long B, Long DA, Tannenbaum L, Koyfman A. An emergency medicine approach to troponin elevation due to causes other than occlusion myocardial infarction. Am. J. Emerg. Med. 38(5), 998–1006 (2019). 31864875 10.1016/j.ajem.2019.12.007

[115] Frencken JF, van Baal L, Kappen TH Myocardial injury in critically ill patients with community-acquired pneumonia. A cohort study. Ann. Am. Thorac. Soc. 16(5), 606–612 (2019). 30521759 10.1513/AnnalsATS.201804-286OC

[116] Menéndez R, Méndez R, Aldás I Community-acquired pneumonia patients at risk for early and long-term cardiovascular events are identified by cardiac biomarkers. Chest 156(6), 1080–1091 (2019). 31381883 10.1016/j.chest.2019.06.040

[117] Tersalvi G, Vicenzi M, Calabretta D, Biasco L, Pedrazzini G, Winterton D. Elevated troponin in patients with Coronavirus Disease 2019 (COVID-19): possible mechanisms. J. Cardiac Failure 26(6), 470–475 (2020). 10.1016/j.cardfail.2020.04.009 PMC7166030 32315733

[118] Lippi G, Lavie CJ, Sanchis-Gomar F. Cardiac troponin I in patients with coronavirus disease 2019 (COVID-19): evidence from a meta-analysis. Prog. Cardiovasc. Dis. 63(3), 390–391 (2020). 32169400 10.1016/j.pcad.2020.03.001 PMC7127395

[119] Nie SF, Yu M, Xie T Cardiac troponin I is an independent predictor for mortality in hospitalized patients with COVID-19. Circulation 142(6), 608–610 (2020). 32539541 10.1161/CIRCULATIONAHA.120.048789 PMC7418761

[120] Shi S, Qin M, Shen B Association of cardiac injury with mortality in hospitalized patients with COVID-19 in Wuhan, China. JAMA Cardiol 5(7), 802–810 (2020). 32211816 10.1001/jamacardio.2020.0950 PMC7097841

[121] Chen L, Li X, Chen M, Feng Y, Xiong C. The ACE2 expression in human heart indicates new potential mechanism of heart injury among patients infected with SARS-CoV-2. Cardiovasc. Res. 116(6), 1097–1100 (2020). 32227090 10.1093/cvr/cvaa078 PMC7184507

[122] Maekawa Y, Ouzounian M, Opavsky M, Liu P. Connecting the missing link between dilated cardiomyopathy and viral myocarditis. Circulation 115, 5–8 (2007). 17200452 10.1161/CIRCULATIONAHA.106.670554

[123] Lawson CM. Evidence for mimicry by viral antigens in animal models of autoimmune disease including myocarditis. Cellu. Mol. Life Sci. 57(4), 552–560 (2020). 10.1007/PL00000717 PMC11146955 11130455

[124] Driggin E, Madhavan MV, Bikdeli B Cardiovascular considerations for patients, health care workers, and health systems during the COVID-19 pandemic. J. Am. Coll. Cardiol. 75(18), 2352–2371 (2020). 32201335 10.1016/j.jacc.2020.03.031 PMC7198856

[125] Chapman AR, Bularga A, Mills NL. High-sensitivity cardiac troponin can be an ally in the fight against COVID-19. Circulation 141(22), 1733–1735 (2020). 32251612 10.1161/CIRCULATIONAHA.120.047008

[126] Kell DB, Pretorius E. Serum ferritin is an important inflammatory disease marker, as it is mainly a leakage product from damaged cells. Metallomics 6(4), 748–773 (2014). 24549403 10.1039/c3mt00347g

[127] Enomoto N, Oyama Y, Enomoto Y Prognostic evaluation of serum ferritin in acute exacerbation of idiopathic pulmonary fibrosis. Clin. Respir. J. 12(8), 2378–2389 (2020). 10.1111/crj.12918 29873202

[128] Vargas-Vargas M, Cortés-Rojo C. Ferritin levels and COVID-19. Revista Panamericana de Salud Pública 44, e72 (2020). 32547616 10.26633/RPSP.2020.72 PMC7286435

[129] Zhou B, She J, Wang Y, Ma X. Utility of ferritin, procalcitonin, and C-reactive protein in severe patients with 2019 novel coronavirus disease. Res. Square (2020). https://www.researchsquare.com/article/rs-18079/v1

[130] Zhou F, Yu T, Du R Clinical course and risk factors for mortality of adult inpatients with COVID-19 in Wuhan, China: a retrospective cohort study. Lancet 395(10229), 1054–1062 (2020). 32171076 10.1016/S0140-6736(20)30566-3 PMC7270627

[131] Mehta P, McAuley DF, Brown M, Sanchez E, Tattersall RS, Manson JJ. COVID-19: consider cytokine storm syndromes and immunosuppression. Lancet 395(10229), 1033–1034 (2020). 32192578 10.1016/S0140-6736(20)30628-0 PMC7270045

[132] Ji D, Zhang D, Xu J Prediction for progression risk in patients with COVID-19 pneumonia: the CALL score. Clin. Infect. Dis. 71(6), 1393–1399 (2020). 32271369 10.1093/cid/ciaa414 PMC7184473

[133] Rosário C, Zandman-Goddard G, Meyron-Holtz EG, D'Cruz DP, Shoenfeld Y. The hyperferritinemic syndrome: macrophage activation syndrome, Still's disease, septic shock and catastrophic antiphospholipid syndrome. BMC Med. 11(1), 1–1 (2013). 23968282 10.1186/1741-7015-11-185 PMC3751883

[134] Feld J, Tremblay D, Thibaud S, Kessler A, Naymagon L. Ferritin levels in patients with COVID-19: a poor predictor of mortality and hemophagocytic lymphohistiocytosis. Int. J. Lab. Hematol. 42(6), 773–779 (2020). 32790918 10.1111/ijlh.13309 PMC7436675

[135] Cheng Y, Luo R, Wang K Kidney disease is associated with in-hospital death of patients with COVID-19. Kidney Int. 97(5), 829–838 (2020). 32247631 10.1016/j.kint.2020.03.005 PMC7110296

[136] Cui X, Yu X, Wu X Acute kidney injury in patients with the coronavirus disease 2019: a multicenter study. Kidney Blood Press. Res. 45(4), 612–622 (2020). 32712607 10.1159/000509517 PMC7445371

[137] Chu K, Tsang W, Tang C Acute renal impairment in coronavirus-associated severe acute respiratory syndrome. Kidney Int. 67(2), 698–705 (2005). 15673319 10.1111/j.1523-1755.2005.67130.x PMC7112337

[138] Chen N, Zhou M, Dong X Epidemiological and clinical characteristics of 99 cases of 2019 novel coronavirus pneumonia in Wuhan, China: a descriptive study. Lancet 395(10223), 507–513 (2020). 32007143 10.1016/S0140-6736(20)30211-7 PMC7135076

[139] Li Z, Wu M, Yao J Caution on kidney dysfunctions of COVID-19 patients. SSRN Electronic J. 41(1), 1–90 (2020).

[140] Zou X, Chen K, Zou J, Han P, Hao J, Han Z. Single-cell RNA-seq data analysis on the receptor ACE2 expression reveals the potential risk of different human organs vulnerable to 2019-nCoV infection. Front. Med. 12, 1–8 (2020). 10.1007/s11684-020-0754-0 PMC7088738 32170560

[141] Qian J, Wang B, Liu B. Acute kidney injury in the 2019 novel coronavirus disease. Kidney Dis. 6(5), 318–323 (2020). 10.1159/000509086 PMC7360511 32742978

[142] Vincent JL, Moreno R, Takala J The SOFA (Sepsis-related Organ Failure Assessment) score to describe organ dysfunction/failure. Intensive Care Med. 22, 707–710 (1996). 8844239 10.1007/BF01709751

[143] Nadim MK, Forni LG, Mehta RL COVID-19-associated acute kidney injury: consensus report of the 25th Acute Disease Quality Initiative (ADQI) Workgroup. Nat. Rev. Nephrol. 15, 1–8 (2020). 10.1038/s41581-020-00356-5 PMC7561246 33060844

[144] Tang YW, Schmitz JE, Persing DH, Stratton CW. Laboratory diagnosis of COVID-19: current issues and challenges. J. Clin. Microbiol. 58, 6 (2020). 10.1128/JCM.00512-20 PMC7269383 32245835

[145] Li X, Geng M, Peng Y, Meng L, Lu S. Molecular immune pathogenesis and diagnosis of COVID-19. J. Pharmaceut. Anal. 10(2), 102–108 (2020). 10.1016/j.jpha.2020.03.001 PMC7104082 32282863

[146] Coronavirus Disease 2019 (COVID-19) – symptoms. centers for disease control and prevention (2020). https://www.cdc.gov/coronavirus/2019-ncov/symptoms-testing/symptoms.html

[147] Moein S, Hashemian S, Mansourafshar B, Khorram-Tousi A, Tabarsi P, Doty R. Smell dysfunction: a biomarker for COVID-19. Int. Forum Allergy Rhinol. 10(8), 944–950 (2020). 32301284 10.1002/alr.22587 PMC7262123

[148] Defining the burden of olfactory dysfunction in COVID-19 patients. European Review (2020). https://www.europeanreview.org/article/20797 10.26355/eurrev_202004_20797 32329813

[149] Li K, Wohlford-Lenane C, Perlman S Middle East respiratory syndrome coronavirus causes multiple organ damage and lethal disease in mice transgenic for human dipeptidyl peptidase 4. J. Infect. Dis. 213(5), 712–722 (2015). 26486634 10.1093/infdis/jiv499 PMC4747621

[150] Nancy Walsh. Low Vitamin D Tied to COVID-19 Severity (2020). https://www.medpagetoday.com/meetingcoverage/asbmr/88586

[151] Liu D, Tian Q, Zhang J Association of 25 hydroxyvitamin D concentration with risk of COVID-19: a Mendelian randomization study. medRxiv (2020). https://www.medrxiv.org/content/10.1101/2020.08.09.20171280v2 10.3967/bes2021.104 PMC8485511 34530967

[152] Ma H, Zeng W, He H Serum IgA, IgM, and IgG responses in COVID-19. Cell. Mol. Immunol. 17(7), 773–775 (2020). 32467617 10.1038/s41423-020-0474-z PMC7331804

[153] Liu X, Wang J, Xu X, Liao G, Chen Y, Hu CH. Patterns of IgG and IgM antibody response in COVID-19 patients. Emerg. Microbes Infect. 9(1), 1269–1274 (2020). 32515684 10.1080/22221751.2020.1773324 PMC7448841

[154] Lauc G, Sinclair D. Biomarkers of biological age as predictors of COVID-19 disease severity. Aging 12(8), 6490 (2020). 32268300 10.18632/aging.103052 PMC7202497

[155] Hillary RF, Stevenson AJ, McCartney DL Epigenetic clocks predict prevalence and incidence of leading causes of death and disease burden. BioRxiv (2020). https://www.biorxiv.org/content/10.1101/2020.01.31.928648v1 10.1186/s13148-020-00905-6 PMC7394682 32736664

